# Specific IgE responses in patients allergic to goat’s milk but tolerant to cow’s milk: involvement of minor differences in primary structure between caprine and bovine caseins

**DOI:** 10.1186/2045-7022-1-S1-O9

**Published:** 2011-08-12

**Authors:** Sandrine Ah-Leung, Fany Blanc, Stéphane Hazebrouck, Karine Adel-Patient, Evelyne Paty, Pierre Scheinmann, Jean-Michel Wal, Hervé Bernard

**Affiliations:** 1 INRA, Unité d'Immuno-Allergie Alimentaire, Gif-sur-Yvette, France; 2 Hôpital Necker Enfants Malades, Paris, France

## Background

Allergy to goat’s milk (GM) proteins in patients tolerant to cow’s milk (CM) is nowadays often observed whereas CM allergy was generally associated with a cross allergy to GM. We aimed to analyse the specific IgE response in patients allergic to GM but tolerant to CM and to compare this response to that observed in patients allergic to both milks.

## Methods

β-Lactoglobulin, whole casein and its four different fractions, i.e. αs1-, αs2-, β- and κ-caseins, were isolated from raw CM and GM. Purified β-caseins were subjected to a mild proteolysis by plasmin which generated 3 peptides, i.e. f(1-28), f(29-107) and f(108-207/9). Synthetic peptides partially recovering the N-terminal f(29-107) part of the caprine β-casein were also produced. Immunoreactivity of the purified proteins and peptides was assessed by IgE binding studies using sera from 12 GM-allergic patients tolerant to CM and 10 CM-allergic patients. The capacity of bovine and caprine milk proteins to induce the degranulation of humanized rat mast cells passively sensitized with human specific IgE was also evaluated.

## Results

In patients allergic to CM the IgE-immunoreactivity of homologous proteins and peptides from either CM or GM are positively correlated. In contrast, all bovine proteins and related peptides were poorly IgE-immunoreactive in patients allergic to GM but tolerant to CM. These patients showed a specific IgE response restricted to the caprine αs1-,αs2-and β-caseins. The fragment f(29-107) from goat β-casein and to a lesser extent , the complementary one f(108-207) were highly immunoreative. The IgE response to goat β-casein is partly directed against the short peptide f(59-79) which differs from its bovine counterpart by only 2 amino acids substitutions.

## Conclusion

Allergy to GM in patients tolerant to CM is associated with an IgE response specific to caprine caseins without any cross reactivity to bovine counterparts despite sequence homology of ca. 90%. As observed with peptides derived from β-casein, the lack of cross-reactivity between bovine and caprine caseins can be explained by few modifications in the primary structure of the proteins.

